# Genome survey sequencing of common vetch (*Vicia sativa* L.) and genetic diversity analysis of Chinese germplasm with genomic SSR markers

**DOI:** 10.1007/s11033-021-06875-z

**Published:** 2021-11-06

**Authors:** Lin Ma, Xiao Wang, Min Yan, Fang Liu, Shuxing Zhang, Xuemin Wang

**Affiliations:** 1 Institute of Animal Sciences, Chinese Academy of Agricultural Sciences, Beijing, 100193 China; 2 National Animal Husbandry Station, Ministry of Agriculture Sciences, Beijing, 100125 China

**Keywords:** Chinese common vetch, Genome survey, SSR, Genetic diversity

## Abstract

**Background:**

Common vetch (*Vicia sativa* L.) is an annual legume with excellent suitability in cold and dry regions. Despite its great applied potential, the genomic information regarding common vetch currently remains unavailable.

**Methods and results:**

In the present study, the whole genome survey of common vetch was performed using the next-generation sequencing (NGS). A total of 79.84 Gbp high quality sequence data were obtained and assembled into 3,754,145 scaffolds with an N50 length of 3556 bp. According to the K-mer analyses, the genome size, heterozygosity rate and GC content of common vetch genome were estimated to be 1568 Mbp, 0.4345 and 35%, respectively. In addition, a total of 76,810 putative simple sequence repeats (SSRs) were identified. Among them, dinucleotide was the most abundant SSR type (44.94%), followed by Tri- (35.82%), Tetra- (13.22%), Penta- (4.47%) and Hexanucleotide (1.54%). Furthermore, a total of 58,175 SSR primer pairs were designed and ten of them were validated in Chinese common vetch. Further analysis showed that Chinese common vetch harbored high genetic diversity and could be clustered into two main subgroups.

**Conclusion:**

This is the first report about the genome features of common vetch, and the information will help to design whole genome sequencing strategies. The newly identified SSRs in this study provide basic molecular markers for germplasm characterization, genetic diversity and QTL mapping studies for common vetch.

**Supplementary Information:**

The online version contains supplementary material available at 10.1007/s11033-021-06875-z.

## Introduction

Common vetch (*Vicia sativa* L.) is one kind of self-pollinating annual legume with excellent suitability in cold and dry regions, such as western Asia and northern Africa [1]. As an inexpensive and rich source of protein, common vetch grows quickly and is normally used for feeding livestock on account of its high digestibility [2]. In addition, common vetch can also be used as a green manure crop on idle farmland due to its strong ability to fix nitrogen [3]. Moreover, following with the increasing global population and plant-based protein demands, common vetch could be exploited as a future potential protein source due to its widely adaption in marginal cropping zones with severe drought and cold conditions [4].

However, the genetic information of common vetch remains largely unknown, although some studies have performed transcriptome analysis, genetic diversity analysis with ESTs based on SSR markers, and EMS-induced mutation development [2, 5–7]. Nevertheless, the genomic information regarding common vetch currently remains unavailable, as well as the systematic analysis of Chinese common vetch germplasm. The lack of the reference genome sequence impedes the advances in functional genomics and molecular breeding of this species [8]. Therefore, it is necessary to conduct the genome survey sequencing which would obtain basic knowledge on the genome structure of common vetch, providing a foundation for the further research of this specie.

Recently, the next-generation sequencing (NGS) has been employed as the cost-effective approach to conduct genome survey sequencing [9–11]. Except the basic knowledge of genome structure, the genome survey sequencing will provide a large number of simple sequence repeats (SSRs), which could be developed into molecular markers [12–14]. As the versatile DNA-based markers one kind of markers, SSRs markers showed multiple advantages including co-dominant, more informative and more economical, which were generally used in plant genetic researches, such as population diversity, genetic linkage mapping and evolutionary studies [15, 16]. A larger number of SSR markers are essential for comprehensive genome-wide association studies (GWAS), as well as the quantitative trait locus mapping (QTL) and marker-assisted selection (MAS) [14, 17].

In the present research, we perform the de novo whole genome sequencing of common vetch through NGS, and then assembly to construct a reference genome database. The results showed that the genome of common vetch was estimated to be 1568 Mbp with a heterozygosity of 0.4345%. A total of 76,810 putative SSRs were identified and 58,175 of them were designed as potential SSR markers. In addition, 10 SSRs were validated in 68 Chinese common vetch accessions and suggested the high genetic diversity of Chinese common vetch. Taken together, this study firstly reported the de novo whole genome sequencing of common vetch and firstly analyzed the genetic diversity of Chinese germplasm resources. The genome database and potential SSR markers would provide the foundation for further genomic functional and evolutionary analyses of common vetch, as well as accumulating the development of its molecular breeding.

## Materials and methods

### Plant materials and growth condition

The common vetch (*Vicia sativa*) cv. ‘Lanjian No.1’ from Lanzhou University (Lanzhou, Gansu, China) was chosen for the genome survey. The seeds were planted in 10 cm pots and grown in the greenhouse at 24/22 °C (day/night) temperature with 16 h light (380–400 μE/m^2^/s). After growing for three weeks, fresh leaves from one individual plant were collected and quickly frozen in liquid nitrogen for DNA isolation.

A total of 68 common vetch accessions originating from China were used for genetic diversity analysis (Table S1). In addition, 20 common vetch accessions originating from worldwide were used for SSR polymorphism selection (Table S2). The seeds were sterilized in 75% ethanol for 5 min and rinsed with sterile water five times. They were placed on filter paper in dishes and then subsequently cultured in a growth chamber at 25 °C. The eight-day-old seedlings were prepared for DNA extraction.

### DNA extraction and genome sequencing

Total genomic DNA was isolated by using the CTAB method with modifications [18]. DNA concentrations were measured on a Nanodrop (Thermo Fisher Scientific, Waltham, MA). DNA quality was detected on a Qubit (Thermo Fisher Scientific, Waltham, MA). The genomic paired-end library with 300–400 bp short-inserts was constructed and sequenced on an Illumina NovaSeq 6000 (Illumina Inc. San Diego, CA, USA) with PE 150 sequencing methods.

### K-mer analyses and genome size estimation

All of the clean data were used for K-mer analysis using Jellyfish software [19]. Based on the results of K-mer frequency distributions (K-mer = 17), the characteristics of the genome, including genome size, repeat content and heterozygosity rate, were estimated by using GenomeScope [20].

### Genome assembling and guanine plus cytosine (GC) content analysis

SOAPdenovo software was used for genome assembly [21]. In brief, a de Bruijn graph was constructed based on the overlapping relationship reads from SOAPdenovo software, and contigs were output after simplifying the de Bruijn graph. Scaffolds were constructed based on the contigs, and gaps inside the scaffolds were filled by employing GAPCloser. Here, 10-kb nonoverlapping sliding windows along the assembled sequence were hired to calculate the average GC sequencing depth [22].

### Genomic microsatellite identification

The MIcroSAtellite (MISA) software (http://pgrc.ipk-gatersleben.de/misa/misa.html) was employed to detect the genomic SSRs. The search parameters were respectively set for identifying various types of SSRs, including Di-, Tri-, Tetra-, Penta- and Hexa-nucleotide SSR motifs with a minimum of 6, 5, 4, 4 and 4 repeats, respectively. Primer 3 software were used for primers designing for each SSR locus, with the following parameters: 18–25 primer size, 90–250 bp product size, 70% GC content, and annealing temperature of 55–65 °C.

### SSR genotyping

For SSR polymorphism selection, a total of 20 common vetch accessions were used (Table S2). Firstly, sixty pairs of putative SSR primers were randomly selected to test whether they harbored polymorphisms among 20 common vetch varieties with multiply phenotypes by the methods of non-denaturing polyacrylamide gel electrophoresis (PAGE). Then ten SSR were selected for further study.

The selected 10 pairs SSR primers with additional different fluorescent probes on forward primers were used for polymorphism screening of 68 common vetch accessions in China (Table S3). The PCR was conducted in a 20 μL reaction system containing 20 ng genomic DNA, 10 μL 2 × Taq Master Mix (Genestar, Beijing, China) and 0.5 μL each of the forward and reverse primers. The PCR parameters were as follows: 94 °C for 5 min; 35 cycles: 94 °C for 30 s, 58 °C for 30 s, 72 °C for 45 s; the final extension at 72 °C for 7 min. The PCR products were diluted five times and then resolved in an ABI3130xl Genetic Analyzer (Applied Biosystems, CA, USA). Fragments size and data analysis were determined by using mapmaker 5.0 software.

### Genetic diversity analysis

The allelic diversity and genetic variation parameters including the number of different alleles (Na) and effective alleles (Ne), the index of observed heterozygosity (Ho), expected heterozygosity (He), Shannon’s information index (I) and the polymorphism information content (PIC) were calculated by fragment size in GenAlEx 6.5 software [23]. The genetic diversity among the 68 accessions was determined by using dissimilarity analysis and representation for windows (DARwin) software. The dendrogram was generated by using the UPGMA phylogenetic cluster analysis [24].

## Results

### Genome sequencing and sequence assembly

**Fig. 1:**
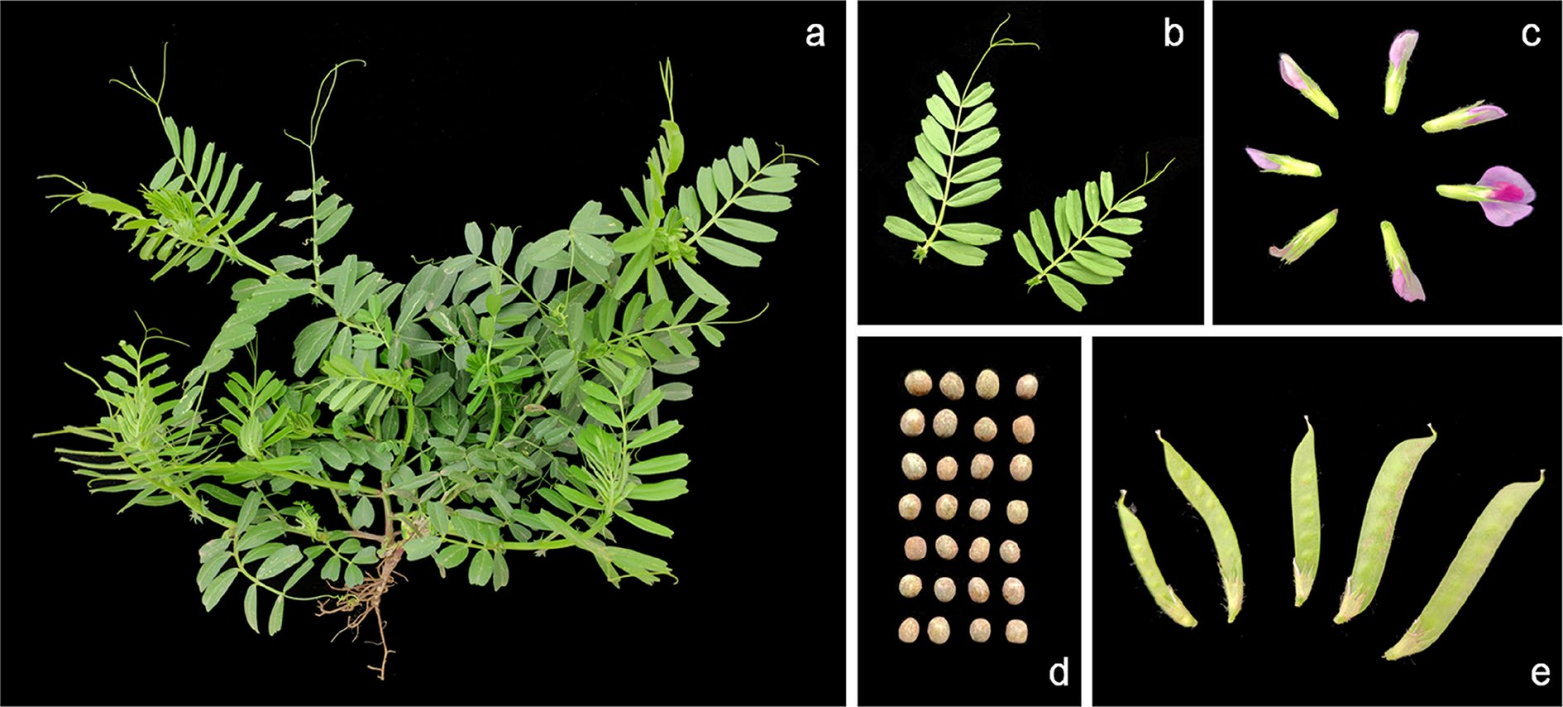
The morphological characteristics of common vetch cv “Lanjian No.1”. **a** The plant. **b** The leaves. **c** The flowers. **d** The seeds. **e** The seedpods

To avoiding the influence the potential heterozygous, we extracted DNA from the single plant leaves of common vetch for libraries constructing (Fig. 1). After filtering the low quality data, we obtained approximately 79.84 Gbp of high-quality data from the sequencing library, which were approximately 51 times of the estimated genome size. The Q20 and Q30 of the obtained data were greater than 97% and 92%, indicating the reliable of the genome survey sequencing. We then de novo assembled (K-mer = 75) all of the high quality data by using the de Bruijn graph-based SOAPdenovo software. A total of 4,227,942 raw contigs were obtained, and the total length of raw contigs was 1,475,990,986 bp and the contig N50 length of 1245 bp (Table 1). Finally, the assembled common vetch genome consisted of 3,754,145 scaffolds which had a total length of 1,516,858,186 bp, and the scaffold N50 length of 3556 bp (Table 1).

**Table 1:** Information of the assembled genome sequences of common vetch

Contigs	
Number of sequences	4,227,942
Total length (bp)	1,475,990,986
Max length (bp)	38,860
N50 length (bp)	1245
N90 length (bp)	115

Scaffolds	
Number of sequences	3,754,145
Total length (bp)	1,516,858,186
Max length (bp)	89,299
N50 length (bp)	3556
N90 length (bp)	116
GC content	35.94%

### Genomic characteristics

**Fig. 2:**
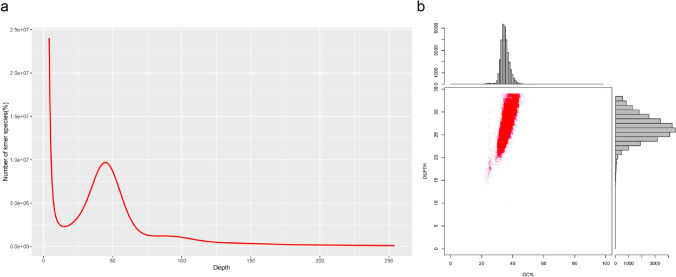
K-mer frequency distribution at K-mer = 17 depth and GC content and depth correction analysis. **a** The estimated genome size of common vetch was determined based on the following formula: genome size = K-mer depth. The x-axis is depth and y-axis represents the frequency at the particular depth divided by the total frequency of all depths. **b** The x-axis represents the GC content and the y-axis is the sequence depth. The distribution of the sequence depth is on the right side, while the distribution of the GC content is at the top

The peak K-mer depth and the number of K-mers were calculated as 45 and 70, 575, 281,718, respectively, based on the K-mer analysis (K-mer = 17). The genome size of common vetch was estimated at 1568 Mbp, while the heterozygosity rate of this genome was 0.4345%, indicating that common vetch was a self-pollinating species (Fig. 2a).

In order to investigate the guanine plus cytosine (GC) content of the common vetch genome, we built a scatterplot graph by using scaffolds larger than 500 bp, elucidating the information on sequencing data bias (Fig. 2a). The results showed that the GC content of the common vetch genome was 35%, which was consistent with the main peak in the scatterplot graph. Moreover, we also noticed that the confidence area (shown in red) was around the peak at 35, suggesting that the DNA sample for genome survey sequencing was not polluted by DNA from other species.

### Genomic SSR markers development

The assembled scaffolds were employed for genomic SSR search via the MISA software (http://pgrc.ipk-gatersleben.de/misa/misa.html). A total of 76,810 putative SSRs were identified from 58,373 isoforms and 12,050 isoforms contained more than one SSR. Among the identified putative SSRs, 4932 SSRs were present in compound formation. We found that the most abundant SSR type was Dinucleotide, accounting for 44.94% of the total SSRs, followed by Tri- (35.82%), Tetra- (13.22%), Penta- (4.47%) and hexa nucleotide (1.54%) SSRs (Fig. S1). The density of SSRs identified in the assembled common vetch genome was one SSR per 20.41 kb.

The SSRs were categorized by their repeat motifs. The most abundant repeats were AG/CT (17.29%) and AC/GT (15.54%), followed by AT/AT (12.02%), AAC/GTT (10.07%), AAT/ATT (9.93%) and AAG/CTT (9.37%), and AAAT/ATTT (4.95%). The most abundant pentanucleotide repeats were AAAAT/ATTTT (1.27%) and AAACC/CGTTT (0.79%) (Fig. S2). Furthermore, we designed primers for 58,175 SSRs by using Primer 3.0 software. The detailed primers are shown in Table S4.

### Genetic diversity and cluster analysis of Chinese common vetch

Ten SSR markers with polymorphisms were selected randomly to investigate genetic diversity of 68 Chinese common vetch accessions. In total, we obtained 76 alleles from the 10 SSR loci (Table 2). For each SSR loci, the number of different alleles (Na) and the effective number of alleles (Ne) were ranged from 3 (SSR-12) to 16 (SSR-13) and 1.2786 (SSR-5) to 6.1286 (SSR-13), respectively. The mean Na and Ne were 7.6 and 3.4905. The index of observed heterozygosity (Ho) and expected heterozygosity (He) ranged from 0 (SSR-12) to 0.1765 (SSR-10) and 0.3195 (SSR-5) to 0.8430 (SSR-13), with the average of 0.0632 and 0.6438, respectively. The polymorphism information content (PIC) ranged from 0.217802 (SSR-5) to 0.836845 (SSR-13) with an average of 0.639076. Other parameter, such as Shannon’s information index (I), ranged from 0.5341(SSR-5) to 2.1759 (SSR-5) with an average of 1.3387. Together, we noticed that SSR-13 harbored the highest polymorphism, followed by SSR-14, and the polymorphism of SSR-5 was the lowest (Table 2). These results suggested that the 68 common vetch accessions from China harbored high genetic diversity.

**Table 2:** Diversity statistic from 10 SSR tested in Chinese common vetch accessions (n = 68)

Locus	Na	Ne	I	Ho	He	PIC
SSR-1	4	3.0552	1.1539	0.0294	0.6777	0.672682
SSR-2	5	2.874	1.0305	0.0735	0.5670	0.562873
SSR-3	6	2.7956	1.1717	0.0735	0.6471	0.642264
SSR-5	6	1.2786	0.5341	0.0294	0.3195	0.217802
SSR-9	10	3.6918	1.7656	0.0147	0.7345	0.729144
SSR-10	5	2.3289	0.9759	0.1765	0.5748	0.570627
SSR-11	11	5.318	1.9084	0.0441	0.8180	0.811961
SSR-12	3	2.0588	0.7595	0.0000	0.5180	0.514268
SSR-13	16	6.1286	2.1759	0.0735	0.8430	0.836845
SSR-14	10	5.9626	1.9111	0.1176	0.8385	0.83229
MEAN	7.6	3.4905	1.3387	0.0632	0.6438	0.639076
SD	4.0332	1.7276	0.5584	0.0528	0.1897	0.188304

*Na* observed number of alleles, *Ne* effective number of alleles, *I* Shannon’s information index, *Ho* observed heterozygosity, *He* expected heterozygosity

**Fig. 3:**
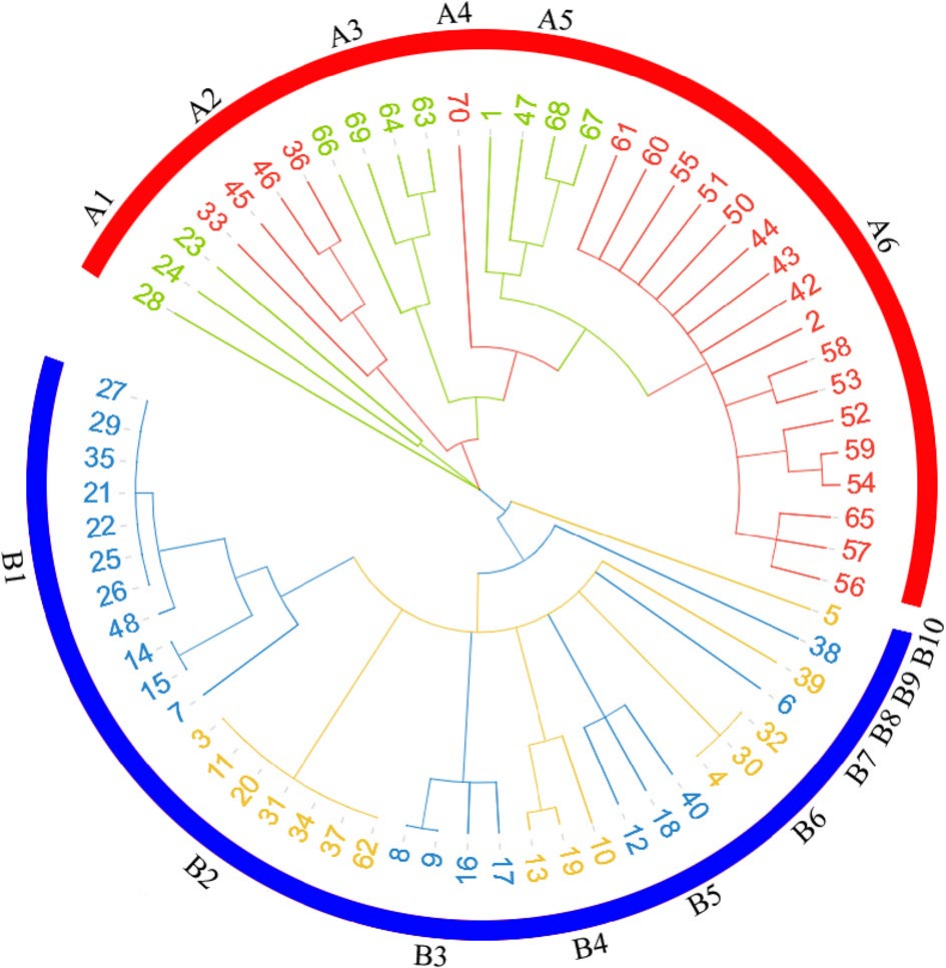
Cluster diagram for 68 individuals of Chinese common vetch by UPGMA method

In addition, we also constructed the hierarchical tree of the Chinese common vetch accessions based on dissimilarity data, to infer phylogenetic relationships among these 68 accessions. Unweighted neighbor-joining analysis resulted in a dendrogram with two main subgroups (A and B) with 6 and 10 clusters, respectively (Fig. 3). In detail, subgroup A consisted of 33 accessions and most of them were wild accessions or landraces; in contrast, subgroup B was composed of 35 accessions but only 17 of them were wild accessions (4) and landraces (13). In addition, we hardly connected the clusters with their original places, suggesting that more markers should be hired in further population structure analysis.

## Discussion

With the increasing global population, global protein demand is predicted to notably increase by 50% by 2050 [25]. The extreme climate changes resulting from global warming and the urgent demand for protein have led to the search for suitable species which provide sustainable protein resources [26]. Common vetch (*Vicia sativa L.*), with excellent drought and cold tolerance, could be exploited to cope with the requirements of humans and livestock [5, 8]. However, the high r-glutamyl-b-cyano-alanine (GBCA) content in common vetch seeds has restricted its application in agriculture, and the traditional strategies have failed to breed common vetch with no GBCA [4].

Modern biotechnology-based high quality references have shown promise in accelerating crop improvement [27]. With the development of functional genomics research, we can obtain the toxin-free common vetch with the use of through modern biotechnology, including over-expression or RNA interference of the candidate genes, or editing the genome by CRISPR-Cas (clustered regularly interspaced short palindromic repeats—Cas protein). These biotechnology methods have already been successfully applied in other major crops, such as rice, wheat, maize and barley [28].

In the present study, we performed a genome survey of common vetch through NGS and obtained the genome information, including genome size, heterozygosity and GC content (Table 1, Fig. 1). This approach has been used to analyses a number of plant genomes, such as pistachio [14], *Acer truncatum* Bunge [29], *Akebia trifoliata* [30] and yellow horn [31]. The depth distribution (K-mer = 17) indicated that the genome size of common vetch was 1568 Mbp and the heterozygosity rate was 0.4345% (Fig. 2). The GC content of the common vetch genome was calculated to be 35%. To our knowledge, this report of genomic information of common vetch is the first of its kind, and lays the foundation for future genome assembly and subsequent functional genome research.

The total number of common vetch populations is quite difficult to estimate, as this specie was distributed worldwide. More than 20,000 accessions were kept in the plant genetic resources (PGR) [2]. It is difficult for farmers to directly use or incorporate into breeding programmes due to the large number of germplasm resources. A core collection for common vetch, which consists of 5% total accessions and represents 95% of the genetic diversity, needed to be constructed through evaluating the genetic relationships between accessions [32]. SSRs with significant dominance were used in evaluating genetic diversity in populations, and the genome survey can also provide extremely useful sources for SSR identification [33].

In this study, we identified 76,810 putative SSRs and 58,175 of them were designed as potential SSR markers. Ten of validated SSRs were selected to investigate the genetic diversity of 68 Chinese common vetch accessions with an average PIC was 0.639076 (Table 2). The results showed that the Chinese common vetch accessions harbored high genetic diversity, as well as the efficacy of the SSR markers developed in the present study. The hierarchical tree of 68 Chinese common vetch accessions indicated that these accessions could be clustered into two main subgroups. Further analysis showed that the subgroup A represented the wild and landraces accessions, wherease subgroup B represented the cultivars and commercial variety (Fig. 3). However, 10 SSR markers were largely insufficient for common vetch molecular fingerprint construction and further population structure analysis. Combined with the further research in genome assembly in chromosomal level, the larger number of SSRs identified in this study shows high potential application in construction of the common vetch core collection. Genome-wide association studies (GWAS) in core collection is an effective way for candidate gene identification in functional genome research [2]. Moreover, the larger number of SSR markers are essential for high density linkage map construction in quantitative trait locus (QTL) mapping [34]. The further selection and verification of more SSRs and their corresponding markers should be developed for functional genome research, as well as the molecular marker assisted breeding in the further. Although the study provides the genome features of common vetch, and the information will help to design whole genome sequencing strategies. A further research including chromosomal assembly, gene annotation, SSR mapping, etc. still remains to be analyzed since the complete genomic information is extremely useful.

## Conclusion

In this study, we obtained the first insight into the genome features of common vetch, and the information will help to design whole genome sequencing strategies. The newly identified SSRs were verified in the genetic diversity analysis of Chinese common vetch germplasm resources. This study provides the valuable information for functional genome research in common vetch, as well as the molecular marker assisted breeding in the further.

## Supplementary Information

Below is the link to the electronic supplementary material.

Supplementary file1 (DOCX 521 kb)

Supplementary file2 (XLS 11494 kb)

## Data Availability

The raw data in this research was deposited in the short read archive (SRA) databank (http://www.ncbi.nlm.nih.gov/sra/) and are available under the accession number PRJNA730328.
