# Supplementary material for: Genome survey sequencing of common vetch (Vicia sativa L.) and genetic diversity analysis of Chinese germplasm with genomic SSR markers
Source: Mol Biol Rep. 2021 Nov 6;49(1):313–20. doi: 10.1007/s11033-021-06875-z (PMC8748366; doi:10.1007/s11033-021-06875-z)

**Fig. S1** Distribution of 76,810 putative SSRs in common vetch genome based on repeat type.


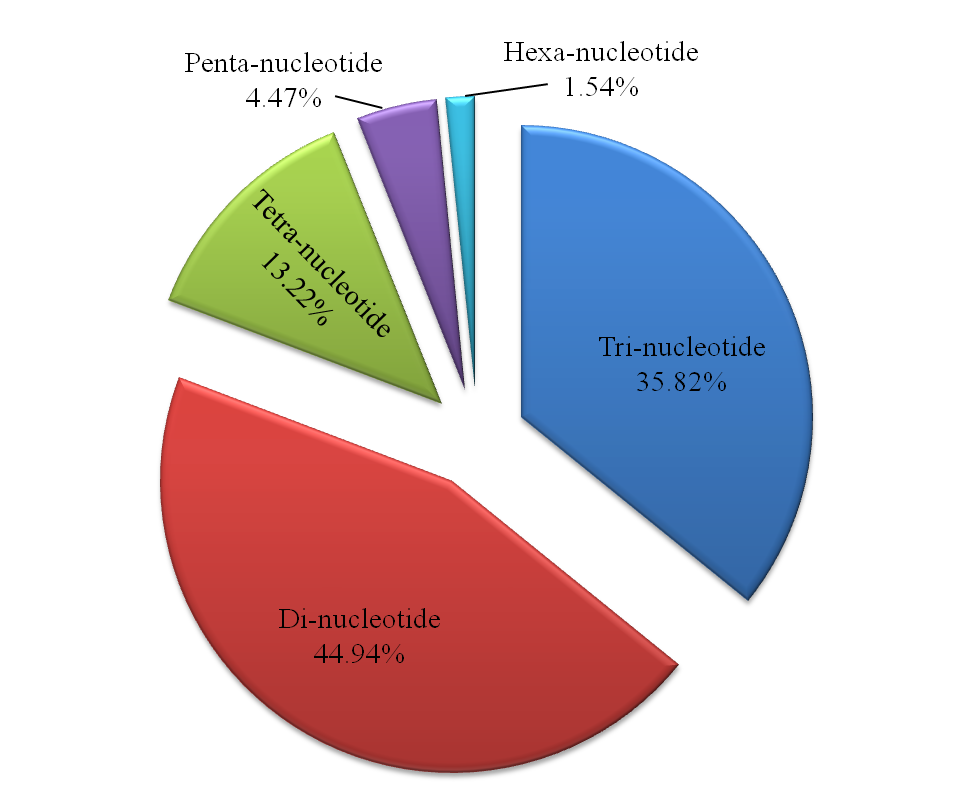


**Fig. S2** The frequency distributions of representative SSR motifs in common vetch. The x-axis and y-axis represent motif type and the motif frequency, respectively.


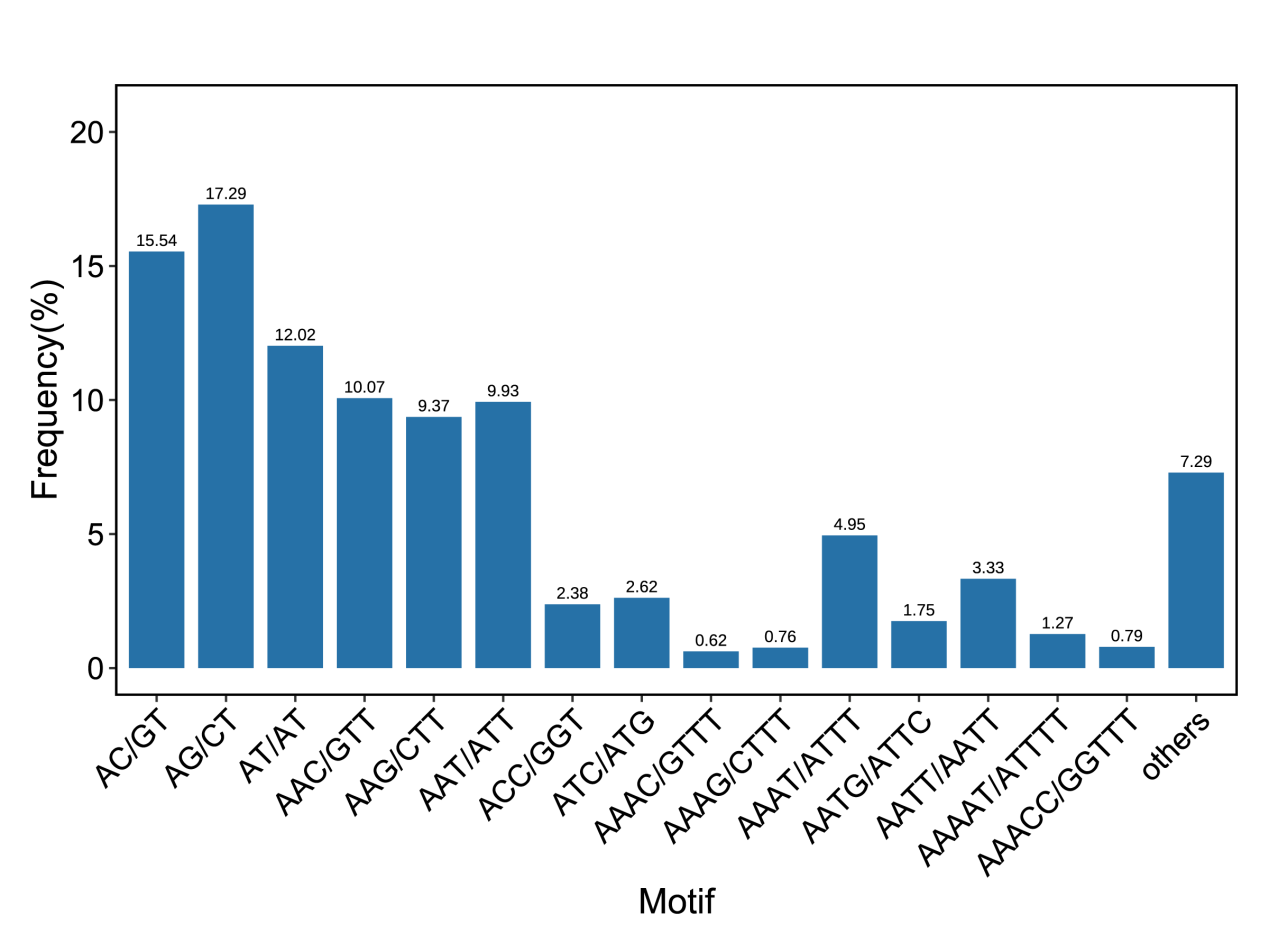

Supplement: Supplementary file 1 — Supplementary file1 (DOCX 521 kb) [file 11033_2021_6875_MOESM1_ESM.docx]
